# A review of effective interventions to improve emotional risk factors of anxiety, stress, depression in infertile and infertile patients undergoing treatment with assisted reproductive techniques

**DOI:** 10.1192/j.eurpsy.2021.1933

**Published:** 2021-08-13

**Authors:** N. Salarian, Z. Hamzehgardeshi, Z. Shahhosseini

**Affiliations:** 1 Sexual And Reproductive Health Research Center, Mazandaran University of Medical Sciences, sari, Iran; 2 Sexual And Reproductive Health Research Center, Department Of Reproductive Health And Midwifery, School Of Nursing And Midwifery, Mazandaran University Of Medical Sciences, Sari, Iran, Mazandaran University of Medical Sciences, Sari, Iran

**Keywords:** mobile application-based intervention, Web based intervention, psychological intervention

## Abstract

**Introduction:**

Infertility is associated with Several negative reaction and emotional problems

**Objectives:**

Review of effective interventions for improving emotional risk factors In infertile and infertile patients undergoing treatment

**Methods:**

A comprehensive narrative review of the studies was conducted. Databases such as Web of Science, Science Direct, Cochrane Library, Scopus, PubMed, including Medline, Clinical key, SID, MAGIRAN were retrieved from August 10 to December 8, 2020, with no time limit. After reviewing the abstract and the full text of the articles in terms of compliance with the purpose of the study, finally, 32 articles were selected for writing. The methodological quality of the articles was assessed based on Cochrane Risk of Bias

**Results:**

Interventions were divided into two subgroups of mind-body, and web-based CBT. mind-body interventions generally shows the anxiety,stress and depression reduction and Possible improvement in pregnancy rate But most of these programs require extensive financial resources The results of web based, showed that using online CBT approach can greatly reduce stress and anxiety, due to increased use of internet, non collaborative,cheap and private treatment of web based interventions, this method can be used as a way along with other treatments to reduce these negative reactions

**Conclusions:**

According to the present study CBT methods, application and Internet-based interventions can be used as appropriate counseling methods in reducing stress, anxiety and improving pregnancy outcomes in infertile patients. This information can be used as a proper source to select appropriate counseling methods for health care providers, midwives and treatment staff involved in infertility patients

**Disclosure:**

No significant relationships.

